# The Hepatotoxicity of Alantolactone and Germacrone: Their Influence on Cholesterol and Lipid Metabolism in Differentiated HepaRG Cells

**DOI:** 10.3390/nu12061720

**Published:** 2020-06-08

**Authors:** Tomáš Zárybnický, Petra Matoušková, Lenka Skálová, Iva Boušová

**Affiliations:** Department of Biochemical Sciences, Faculty of Pharmacy in Hradec Králové, Charles University, 500 05 Hradec Králové, Czech Republic; zarybnto@faf.cuni.cz (T.Z.); matousp7@faf.cuni.cz (P.M.); skaloval@faf.cuni.cz (L.S.)

**Keywords:** alantolactone, germacrone, liver toxicity, metabolic dysregulation, HepaRG cells

## Abstract

The sesquiterpenes alantolactone (ATL) and germacrone (GER) are potential anticancer agents of natural origin. Their toxicity and biological activity have been evaluated using the differentiated HepaRG (dHepaRG) cells, a hepatocyte-like model. The half-maximal inhibitory concentrations of cell viability after 24-h treatment of dHepaRG cells are approximately 60 µM for ATL and 250 µM for GER. However, both sesquiterpenes induce reactive oxygen species (ROS) formation in non-toxic concentrations and significantly dysregulate the mRNA expression of several functional markers of mature hepatocytes. They similarly decrease the protein level of signal transducer and activator of transcription 3 (STAT3), nuclear factor kappa-light-chain-enhancer of activated B cells (NF-κB) and their transcription target, intercellular adhesion molecule 1 (ICAM-1). Based on the results of a BATMAN-TCM analysis, the effects of sesquiterpenes on cholesterol and lipid metabolism were studied. Sesquiterpene-mediated dysregulation of both cholesterol and lipid metabolism was observed, during which these compounds influenced the protein expression of 3-hydroxy-3-methylglutaryl-CoA reductase (HMGCR) and sterol regulatory element-binding protein 2 (SREBP-2), as well as the mRNA expression of *HMGCR*, *CYP19A1*, *PLIN2*, *FASN*, *SCD*, *ACACB,* and *GPAM* genes. In conclusion, the two sesquiterpenes caused ROS induction at non-toxic concentrations and alterations in cholesterol and lipid metabolism at slightly toxic and toxic concentrations, suggesting a risk of liver damage if administered to humans.

## 1. Introduction

Plant secondary metabolites represent a rich library in the search for new chemical structures with medical potential. The sesquiterpenes alantolactone (ATL) and germacrone (GER) are naturally occurring molecules that are studied as potential anticancer agents, especially against breast and liver cancer and glioblastoma cells. ATL, which is one of the major sesquiterpene lactone compounds, is isolated from the roots of Elecampane (*Inula helenium*, Asteraceae). Historically, these roots have been used as a medicine against various ailments such as asthma, cough, bronchitis, tuberculosis, infectious and helminthic diseases [1,2]. GER is a main bioactive constituent found in products containing zedoary oil, which is extracted from *Curcuma zedoaria* Roscoe, a member of the Zingiberaceae family, which is a tropical plant traditionally used as a natural flavoring and medicinal herb for the treatment of dyspepsia, menstrual disorders, flatulence, fever, and cough [3,4]. GER is also a major constituent in essential oils from other curcuma species, such as *Curcuma phaeocaulis* Valeton, also of the Zingiberaceae family, which has antiproliferative activity in breast cancer cells [5]. Owing to its promising anticancer effects, GER has become a target of structural modifications to potentiate its efficacy [6,7].

The mechanism of the potential anticancer activity of both sesquiterpenes involves the induction of reactive oxygen species generation, glutathione depletion, the inhibition of signal transducer and activator of transcription 3 (STAT3) phosphorylation and the B-cell lymphoma 2 (Bcl-2) family of regulator proteins that regulate cell death [8,9,10,11,12,13]. ATL-mediated inhibition of the nuclear factor kappa-light-chain-enhancer of activated B cells (NF-κB) has been described in cancer cells [10,14,15]. However, no change in NF-κB expression was described for GER [16], although the essential oil from *Curcuma phaeocaulis* (with GER as a major constituent) upregulated NF-κβ expression in breast cancer cells [5].

In spite of both compounds having shown a very interesting rate of biological activity, especially in terms of anticancer effect, less attention has been paid to their impact on healthy cells. Therefore, the first aim of this study was to evaluate the effect of ATL and GER on the viability of differentiated HepaRG (dHepaRG) cells, a human hepatocyte-like model. The HepaRG cell line is an original human hepatoma cell line that presents itself as a mixture of hepatocyte islands and biliary epithelial-like cells when cultured in appropriate conditions. Unlike other human hepatocyte cell lines, HepaRG cells depict a closer phenotype to primary human hepatocytes than do HepG2 cells in relation to the expression of drug metabolizing enzymes, drug transporters, and nuclear factors. HepaRG cells are stable in culture for several weeks and responds to various inducers [17,18].

To evaluate the functional changes in dHepaRG cells after ATL and GER exposure, the mRNA expression of functional markers of mature hepatocytes was evaluated; namely: cytochrome P450 3A4 (*CYP3A4*), alpha-1 antitrypsin (*A1AT*), albumin (*ALB*), glucose-6-phosphatase (*G6PC*), and transferrin receptor (*TFRC*) [19].

Since the phenotype of dHepaRG cells is considerably different in comparison to the highly proliferative HepG2 cells [20], we decided to assess the effect of ATL and GER on the above-mentioned transcription factors NF-kB and STAT3 in a time- and concentration-dependent manner. Both transcription factors are regulators of the intercellular adhesion molecule ICAM-1 [21,22,23]. In the liver, this transmembrane glycoprotein is expressed in liver sinusoidal endothelial cells, hepatocytes, Kupffer cells, and hepatic stellate cells, and is further upregulated by inflammatory activation such as stimulation by cytokines tumor-necrosis factor α or interleukin 1β. Moreover, ICAM-1 likely plays a major role in metastatic progression [24]. The expression changes of ICAM-1 after ATL and GER treatments of dHepaRG cells were therefore assessed.

To search for new molecular targets of tested sesquiterpenes, the PubChem CID numbers of ATL and GER were used to analyze their function with the bioinformatic analysis tool BATMAN-TCM [25] to identify potential genes or proteins involved. An analysis of targets common for both sesquiterpenes led us to further focus on cholesterol metabolism and the major regulating enzyme of mevalonate pathway 3-hydroxy-3-methylglutaryl-CoA reductase (HMGCR). Since GER has been described to attenuate hyperlipidemia in high-fat diet obese mice [26] as well as to inhibit adipogenesis and stimulate lipolysis in 3T3-L1 preadipocytes [27], the changes in the expression of major genes responsible for de novo lipogenesis, triglyceride synthesis, and lipid sequestration in dHepaRG cells after ATL and GER treatment were evaluated.

## 2. Materials and Methods

ATL was obtained from Extrasynthese (Lyon, France) and GER was obtained from Santa Cruz Biotechnology (Heidelberg, Germany). Both the tested compounds were pre-dissolved in DMSO. The final concentration of dimethylsulfoxide (DMSO) in culture medium did not exceed 0.1%. All the other chemicals were obtained from Sigma Aldrich (Prague, Czech Republic) unless stated otherwise.

### 2.1. Cell Culture

Cryopreserved HepaRG cells were obtained from Biopredic International (Rennes, France). Undifferentiated cells were seeded according to the supplier protocols in 96-well plates for the viability assays, and in 6-well plates for the mRNA and western blot measurements. The cells were cultured for two weeks in Williams E Medium with GlutaMAX™ (Invitrogen, Paisley, UK) supplemented with 10% fetal bovine serum, 2 mM glutamine, 100 IU/mL penicillin, 100 μg/mL streptomycin, 5 μg/mL insulin, and 50 μM hydrocortisone hemisuccinate. The cells were afterwards cultured for another 2-week period in the same medium supplemented with 1.7% DMSO. The medium was replaced every 2–3 days.

The mRNA and protein expression changes were measured after a single administration of ALA and GER. Three concentrations were selected for each compound (non-toxic, non-toxic/slightly toxic and toxic). The cholesterol level of the dHepaRG cells was measured after a multiple exposure of the highest non-toxic concentrations of tested compounds. ATL (30 µM) and GER (100 µM) were administered four times every 24 h. The expression changes of genes and proteins related to cholesterol metabolism were measured in both single and multiple exposure experiments.

### 2.2. Cell Viability

#### 2.2.1. MTT Assay

In the 96-well plates, viability was evaluated using the methylthiazole-tetrazolium (MTT) colorimetric assay. After 24 and 72-h exposure, 25 μL of MTT solution (3 mg of MTT in 1 mL of phosphate buffered saline, PBS) were added to each well. The plates were incubated at 37 °C for an additional 2 h, after which the medium was removed and the formed formazan was dissolved in 50 μL of 0.08 M HCl in isopropanol during 30 min shaking. The absorbance in each well was read at 570 and 690 nm using a spectrophotometer (Tecan Infinite M200, Tecan, Männedorf, Switzerland).

#### 2.2.2. Lactate Dehydrogenase Leakage

Lactate dehydrogenase (LDH) leakage was measured using the Invitrogen™ CyQUANT™ LDH Cytotoxicity Assay Kit according to the manufacturer guidelines using 96-well plates. After 24 and 72-h exposure, the culture medium was collected and diluted ten times by distilled water. Fifty µL of reaction mixture was added to each sample well and mixed. After 30 min of incubation at room temperature, 50 µL of stop solution was added to each well. To determine LDH activity, 680-nm absorbance value (background) was subtracted from the 490-nm absorbance before calculation of % cytotoxicity. Cell lysate was set as 100% of the LDH leakage.

### 2.3. ROS Production

The production of reactive oxygen species (ROS) was measured using 2′,7′-dichlorofluorescin diacetate probe (H2DCFDA). The cells were preincubated with 10 µM H2DCFDA for 30 min in the dark. Afterwards, the cells were washed with PBS and sesquiterpenes were added to a Hank’s Balanced Salt Solution (HBSS) buffer. Fluorescence was measured after 1, 6, 12, and 24 h with excitation and emission wavelengths of 490 nm and 530 nm, respectively, using the Tecan Infinite M200. Hydrogen peroxide (300 µM) served as a positive control.

### 2.4. Target Prediction

The bioinformatic analysis for studying the molecular mechanism of the tested compounds was performed using PubChem CID of ATL (72724) and GER (6436348) entered as search queries in the BATMAN-TCM tool (available at http://bionet.ncpsb.org/batman-tcm/). The following parameter settings were used: Target Prediction’s Score cut off at 20; Target Analysis’s Adjusted *p*-value less than 0.05 [25].

### 2.5. Gene Expression

All treatments were performed in triplicates and every experiment was performed three times. The cells were washed twice with ice cold phosphate-buffered saline (PBS), scrapped and stored at −80 °C until use. Total RNA from cells was isolated using TriReagent according to the manufacturer’s instructions (Biotech, Prague, Czech Republic). The purified RNA was dissolved in 40 µL of diethyl pyrocarbonate (DEPC)-treated water (0.01% DEPC in HPLC water, autoclaved) and stored at −80 °C. The measurement of absorbance at 260 and 280 nm using the NanoDrop ND-1000 UV–VIS Spectrophotometer (Thermo Fisher Scientific, Pardubice, Czech Republic) was used to determine RNA yields and purity. Subsequently, the RNA (4 µg) was treated with 2 U of DNase I (New England Biolabs, Ipswich, MA, USA) in a final volume of 30 µL for 25 min at 37 °C, 1.5 µL of 0.1 M ethylenediaminetetraacetic acid was added and the DNAse was inactivated by heat (5 min at 75 °C). The solution was diluted to a concentration of 0.1 µg/µL. The DNAse treated RNA was stored at −80 °C until further analyses. The first strand cDNA was synthesized from 800 ng of total RNA and 1 µL of 50 µM random hexamers (Generi Biotech, Hradec Kralove, Czech Republic) using ProtoScript II reverse transcriptase (New England Biolabs, Ipswich, MA, USA). After the initial heat denaturation of total RNA (65 °C for 5 min), 4 µL of 5× ProtoScript II RT Reaction Buffer, 2 µL of 10× dithiothreitol (DTT), 2 µL of deoxynucleotide (dNTP) Mix 5 mM, 3.5 µL of H_2_O, and 0.5 µL of ProtoScript II 200 U/µL were added and mixed by pipetting. The reactions (20 µL) were incubated for 10 min at 25 °C, for 50 min at 42 °C, and for 5 min at 80 °C. The obtained cDNAs were diluted 1:6 by DEPC water. The qPCR analyses were carried out using QuantStudio 6 Flex (Applied Biosystems, Foster City, CA, USA) with SYBR green I (Xceed qPCR SG Mix, Institute of Applied Biotechnologies, Prague, Czech Republic) detection according to the manufacturer’s protocol. The samples contained both forward and reverse primers (both 250 nM) and 5 µL of diluted cDNA (list of primers in Table S1). The PCR reactions began with a denaturation step (10 min, 95 °C) followed by 40 cycles of amplification, which consisted of denaturation (10 s, 95 °C), annealing, and extension (40 s, 60 °C). Fluorescence data were recorded at the end of each amplification step. Relative expression levels of the target genes were calculated as fold changes in triplicates for each group using the 2^−ΔΔCt^ method [28] by normalization to the geometric mean of the Ct values of Sdha and Ywhaz, which were used as reference genes.

### 2.6. Western Blotting

All the treatments were performed in triplicates. Protein concentration was measured using the bicinchoninic acid (BCA) protein assay (Sigma Aldrich, Prague, Czech Republic) according to the manufacturer’s instructions. The proteins were loaded onto a 10% sodium dodecyl sulfate (SDS; w/v)–polyacrylamide gel (with 4% stacking gel) and separated by SDS-PAGE electrophoresis. The proteins were transferred onto a nitrocellulose membrane using wet blot (100 V, 90 min). The membrane blocking was performed in a 5% non-fat dry milk/TRIS-buffered saline-Tween-20 (TBS-T) solution at room temperature for 2 h. Incubation with primary antibodies was accomplished overnight at 10 °C. β-actin was used as housekeeping protein (the list of primary antibodies in Table S2). Afterwards, the membrane was washed with 0.05% TBS-T solution for 6 × 5 min, incubated with respective secondary antibodies conjugated with horseradish peroxidase (bovine anti-rabbit (sc2370, Santa Cruz Biotechnology, Santa Cruz, CA, USA) at room temperature for 1.5 h, and rinsed with TBS-T solution for 6 × 5 min. Visualization of protein bands was carried out using a chemiluminescence kit (GE Healthcare, Buckinghamshire, UK).

### 2.7. Cholesterol Level

The cholesterol content in the dHepaRG cells was tested using the Amplex Red Cholesterol Assay kit (Invitrogen) according to the manufacturer’s instructions and as per An et al. [29]. The cells were collected 12 h after the last exposure of the tested compounds in a 4-day exposure with a medium change every 24 h. The cells were rinsed three times in PBS to eliminate residual growth medium. Whole cell extracts were prepared by 0.5 mL of RIPA lysis buffer (0.5% sodium deoxycholate, 0.1% SDS, 1% NP-40, 150 mM NaCl, 1 mM DTT, 50 mM Tris–HCl (pH = 7.4) and phenylmethanesulfonyl fluoride. Fluorescence was measured using a microplate reader (Tecan Infinite M200) at 530 nm excitation and 590 nm emission wavelengths. The total cholesterol (TC) content was determined by measuring the cholesterol concentration following digestion with cholesterol esterase (CE). To measure the free cholesterol (FC), CE was omitted from the assay. Three independent experiments were performed. The values obtained from a cholesterol standard curve were normalized to protein content, measured by a BCA assay.

### 2.8. Statistical Analyses

Data analysis and graphical illustration were performed with GraphPad Prism 8 (GraphPad Software, Inc., San Diego, CA, USA). The experiments were performed in triplicate unless stated otherwise, with results presented as mean ±  SD. Differences among the different culture conditions were detected applying an unpaired Student’s t test. For treatments with the same compound, a one-way Anova with a post hoc Tukey test was applied. Results were considered significant at *p*  <  0.05.

## 3. Results

**Figure 1:**
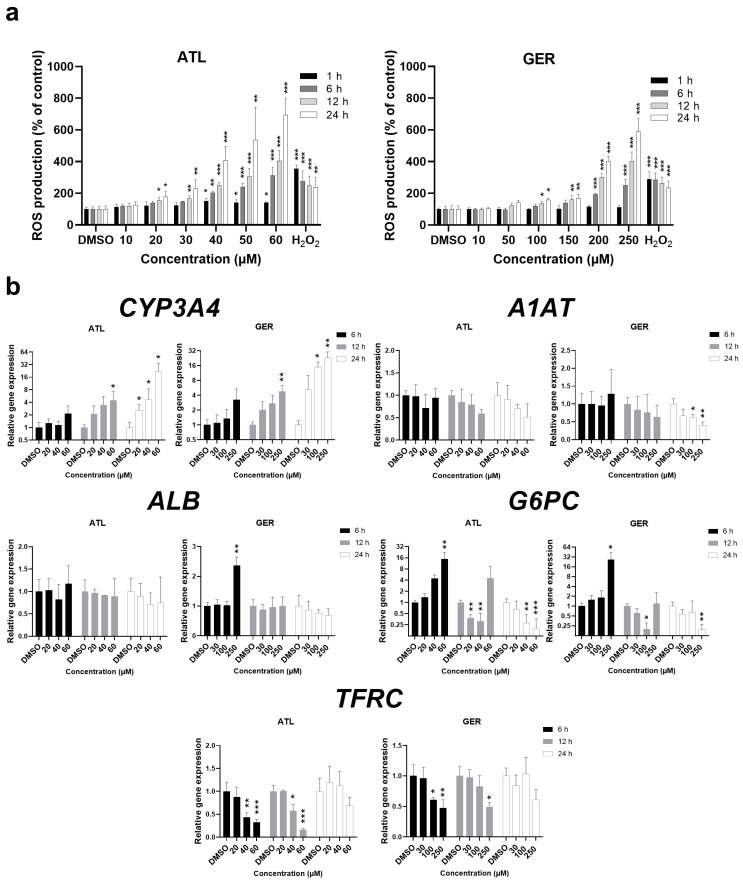
(**a**) Intracellular formation of reactive oxygen species in ATL and GER treated dHepaRG cells, measured using the H_2_-DCFDA (2’,7’-dichlorodihydrofluorescein diacetate) probe in comparison to a positive control of 300 µM H_2_O_2_; (**b**) mRNA expression changes on markers of mature hepatocytes: cytochrome P450 3A4 (*CYP3A4*), alpha-1-antytrypsin (*A1AT*), albumin (*ALB*), glucose-6-phosphatase (*G6PC*), and transferrin receptor (*TFRC*). DMSO, dimethylsulfoxide. Results are expressed as the mean ± SD from three independent experiments. Results statistically significant: *p* < 0.05 (*), *p* < 0.01 (**), *p* < 0.001 (***).

First, the effect of ATL and GER on the cell viability and reactive oxygen species (ROS) production in dHepaRG cells was studied. Cell viability was determined using the MTT method and LDH leakage. A concentration-dependent decrease in the cell viability was observed after ATL- and GER-exposure of the dHepaRG cells for 24 and 72 h (see Figure S1A,B). The determined half-maximal inhibitory concentrations of cell viability (IC_50_) for ATL and GER treatment are presented in Table 1. The production of ROS in the dHepaRG cells exposed to ATL and GER (in various concentrations) for 1, 6, 12, and 24 h was measured using the H2-DCFDA probe. ATL as well as GER exposure caused significant time- and concentration-dependent enhancement of ROS production. The highest concentrations of sesquiterpenes (equal to respective IC_50_) showed higher ROS production than did 300 μM H_2_O_2_ (used as a positive control). The ROS production after ATL and GER treatment are presented in Figure 1A. The functional changes in dHepaRG cells were evaluated by measuring the mRNA expression of mature hepatocyte markers (Figure 1B). Both ATL and GER induced the expression of *CYP3A4*, dysregulated *G6PC* and inhibited *TFRC* expression. These changes were observed even in other than toxic concentrations. On the other hand, GER inhibited the *A1AT* expression after 24 h and induced *ALB* expression after 6 h of exposure.

**Figure 2:**
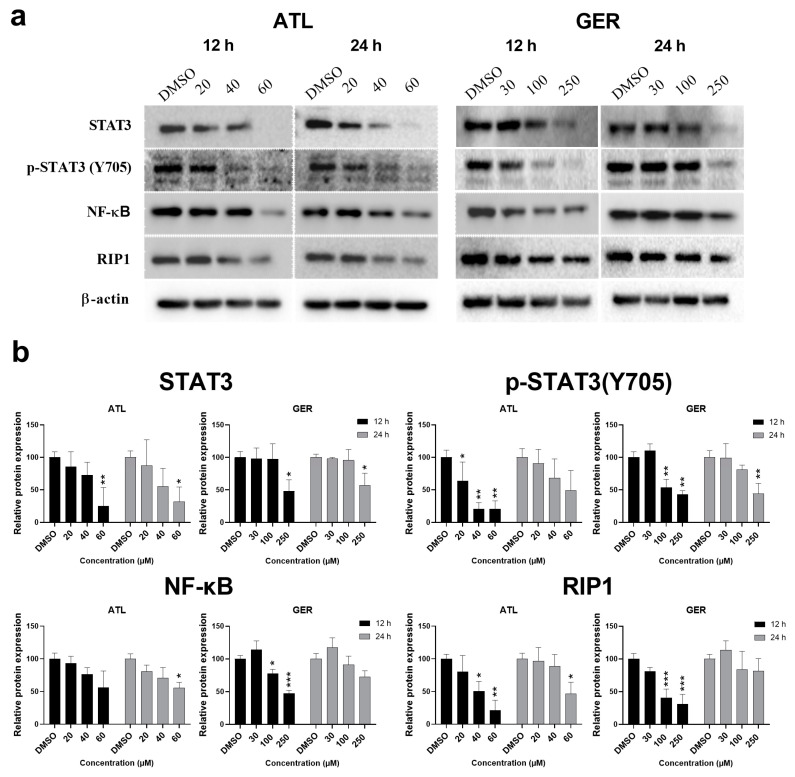
Western blot analysis of transcription factors STAT3 and NF-κB and RIP1 kinase. (**a**) Representative western blots showing expression changes in the studied proteins after ATL and GER treatment. (**b**) Relative protein expression changes in the studied proteins to β-actin. Results are expressed as the mean ± SD from three independent experiments. Results statistically significant: *p* < 0.05 (*), *p* < 0.01 (**), *p* < 0.001 (***).

Our second aim was to assess the effects of both sesquiterpenes on their known targets: transcription factors STAT3 and NF-κB in the dHepaRG cells. The levels of STAT3, phosphorylated p-STAT3(Y705) and NF-κB after 12- and 24-h exposure of dHepaRG cells to ATL and GER in three selected concentrations —non-toxic, non-toxic/slightly toxic, and equal to IC_50_—were evaluated. A 12-h exposure to ATL (in all concentrations tested) or GER (in both higher concentrations) decreased the amount of phosphorylated p-STAT3(Y705). However, both sesquiterpenes (at the highest tested concentration) also decreased the level of total STAT3, unlike in previous experiments published on other cell lines [8,9,13,30,31]. ATL exposure (24-h, the highest concentration) caused significant decrease in the level of NF-κB. Surprisingly, 12-h exposure to GER also led to a decreased level of NF-κB in the dHepaRG cells. As the function of NF-κB is in close relationship with the receptor-interacting kinase 1 (RIP1), its expression was also evaluated, showing a significant decrease after 12-h exposure to both the tested compounds. The RIP1 expression was also decreased by the highest concentration of ATL after 24-h exposure, but not GER. Representative immunoblots and relative protein expression changes are shown in Figure 2.

**Figure 3:**
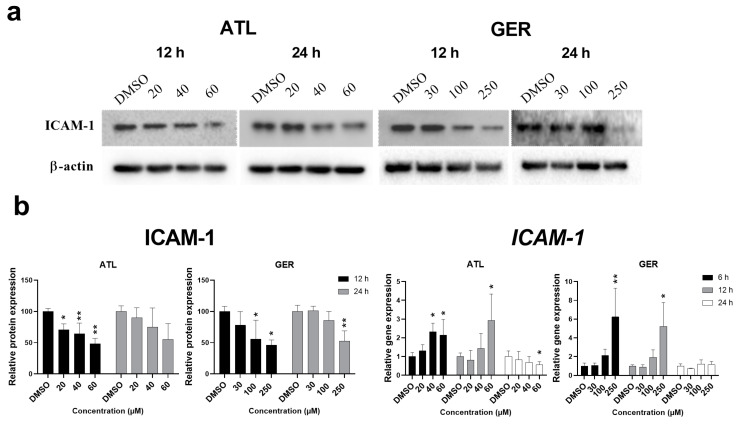
Expression changes in the intercellular adhesion molecule ICAM-1 after ATL and GER treatment of differentiated HepaRG cells. (**a**) Representative western blots showing expression changes in ICAM-1. (**b**) Relative protein expression changes in ICAM-1 to β-actin and relative gene expression changes in *ICAM-1* mRNA by RT-qPCR. Results are expressed as the mean ± SD from three independent experiments. Results statistically significant: *p* < 0.05 (*), *p* < 0.01 (**), *p* < 0.001 (***).

Since both above mentioned transcription factors regulate the expression of intercellular adhesive molecule 1 (ICAM-1), the effect of ATL and GER on its mRNA and protein level was evaluated. The amount of *ICAM-1* mRNA was induced after 12-h and 24-h exposure to ATL (the highest concentration), and after 6-h and 12-h exposure to GER (the highest concentration). In contrast, the amount of ICAM-1 protein decreased after 12 and 24 h of sesquiterpene exposure. Representative immunoblots and relative protein and mRNA expression changes of ICAM-1 are shown in Figure 3.

**Figure 4:**
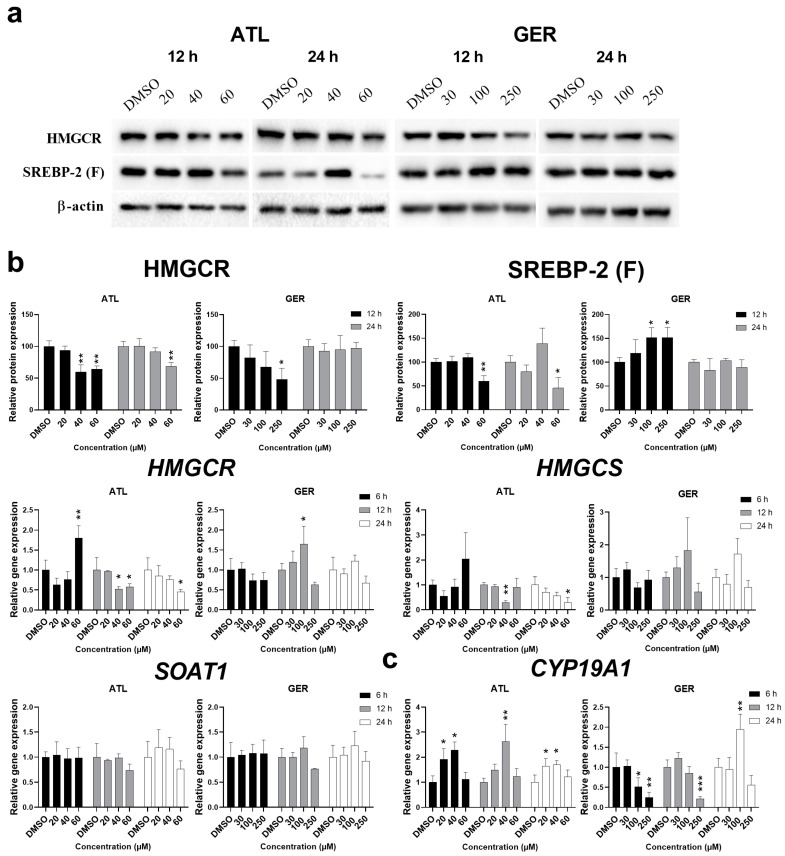
Expression changes in the targets predicted by the BATMAN-TCM after ATL and GER treatment of differentiated HepaRG cells. (**a**) Representative western blots showing expression changes on HMGCR and functional SREBP-2 (SREBP-2 (F)) proteins (**b**) Relative protein expression changes in HMGCR and SREBP-2 (F) after normalization to β-actin and relative gene expression changes in *HMGCR*, *HMGCS* and *SOAT1* mRNA by RT-qPCR. (**c**) Relative gene expression changes in aromatase *CYP19A1* mRNA by RT-qPCR after ATL and GER treatment. Results are expressed as the mean ± SD from three independent experiments. Results statistically significant: *p* < 0.05 (*), *p* < 0.01 (**), *p* < 0.001 (***).

With the aim of determining a novel mechanism of action of the tested sesquiterpenes, the target prediction tool BATMAN-TCM was used. Via the PubChem CID numbers of ATL and GER, 22 and 258 targets were predicted, respectively. Out of these, both sesquiterpenes shared six common targets including 3-hydroxy-3-methylglutaryl-coenzyme A reductase (HMGCR), a major regulatory enzyme involved in cholesterol metabolism, and aromatase (CYP19A1), a key enzyme for estrogen synthesis. It is well known that the liver is a critical organ for maintaining cholesterol and lipid homeostasis, therefore HMGCR, a regulatory enzyme of mevalonate pathway, was of primary interest to us. Total results from the BATMAN-TCM are presented in Table S3, while Venn diagrams of respective results and the list of their common targets are shown in Figure S2. The effect of the tested sesquiterpenes on the BATMAN-TCM predicted targets are presented in Figure 4.

The protein level of HMGCR was measured after 12- and 24-h exposure of dHepaRG cells to ATL or GER. After the treatment of dHepaRG cells with ATL, HMGCR protein amount was shown to decrease both after 12 and 24 h, while GER treatment led to the decrease of HMGCR only after 12-h exposure. Nevertheless, the ATL and GER mediated changes were significant mostly at the highest tested concentrations (equal to respective IC_50_). Since the sesquiterpenes affected HMGCR expression, we analyzed their effect on the level of sterol regulatory element-binding protein 2 (SREBP-2), a major transcription factor responsible for the regulation of most genes in the mevalonate pathway. The functional SREBP-2 (SREBP-2 (F)) protein level was significantly decreased by the highest concentration of ATL. Contrarily, a 12-h exposure to GER led to an increase of SREBP-2 (F), but no change in protein level was observed after 24-h exposure (Figure 4a). In a follow-up procedure, the mRNA levels of *HMGCR* and hydroxymethylglutaryl-CoA synthase (*HMGCS*) were measured after 6, 12, and 24 h of exposure with sesquiterpenes. Due to similar regulation of both genes, similar trends were expected in their expression. Indeed, ATL (the highest concentration) in the dHepaRG cells caused the induction of both genes after 6-h exposure, followed by a decreased level after 12- and 24-h exposure. No change mediated by sesquiterpene exposure was observed for mRNA level in the sterol O-acyltransferase 1 (*SOAT1*) gene (Figure 4b). On the other hand, both sesquiterpenes significantly affected the mRNA level of aromatase (*CYP19A1*), another target predicted by the BATMAN-TCM. The level of *CYP19A1* mRNA was induced by 20 and 40 µM ATL at all the studied intervals, but not by 60 µM ATL. In contrast, GER (at both higher concentrations) decreased the level of *CYP19A1* mRNA after 6- and 12- hour exposure, while 100 µM GER induced the level by nearly two-fold after 24-h exposure (Figure 4c).

**Figure 5:**
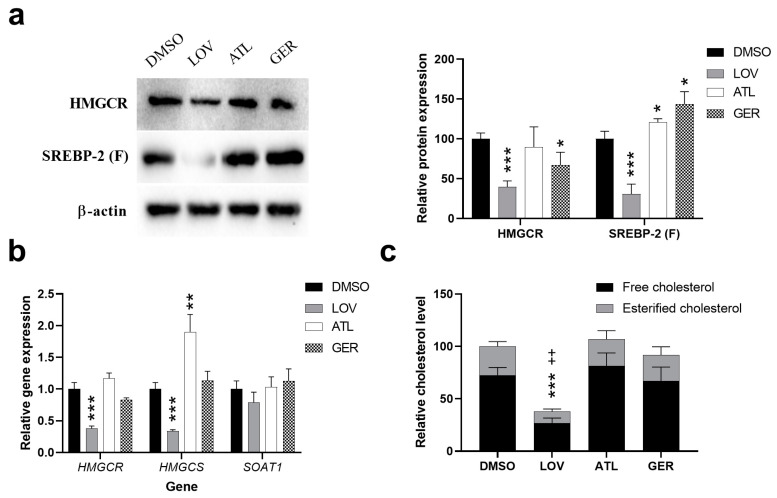
Changes in cholesterol metabolism after a multiple exposure experiment (4-day exposure with medium change every 24 h, cells collected 12 h after the last treatment). (**a**) Representative western blots and relative protein expression changes showing expression changes in HMGCR and SREBP-2 after normalization to β-actin. (**b**) Relative gene expression changes in *HMGCR*, *HMGCS,* and *Soat1* mRNA by RT-qPCR. (**c**) Cholesterol levels in differentiated HepaRG cells after multiple exposure treatment. Results are expressed as the mean ± SD from three independent experiments. * statistical significance for free cholesterol level change; + statistical significance for esterified cholesterol). Results statistically significant: *p* < 0.05 (* or +), *p* < 0.01 (** or ++), *p* < 0.001 (*** or +++).

With the intention of potentiating the effect of ATL and GER on HMGCR and SREBP-2 function and expression, a multiple exposure experiment was performed with the highest non-toxic concentrations of the respective terpenes (30 μM ATL and 100 μM GER). The cells were analyzed 12 h after the last treatment, with a total of four applications of tested compounds every 24 h. A model HMGCR inhibitor, 20 μM lovastatin (LOV) was used as a positive control. However, except for LOV, only GER caused a slight decrease in HMGCR protein level. On the other hand, the amount of SREBP-2 (F) after sesquiterpene treatment was induced, while LOV decreased the SREBP-2 (F) level. LOV decreased significantly the mRNA level of *HMGCR* and *HMGCS*, while ATL induced *HMGCS*. No changes were observed in Soat1 mRNA level after any treatment. The level of free cholesterol and esterified cholesterol in the dHepaRG cells after ATL and GER multiple exposure treatment was analyzed to determine the sesquiterpene mediated changes in cholesterol level. Except for the positive control LOV, no effect was observed. The results of multiple exposure experiment are presented in Figure 5.

**Figure 6:**
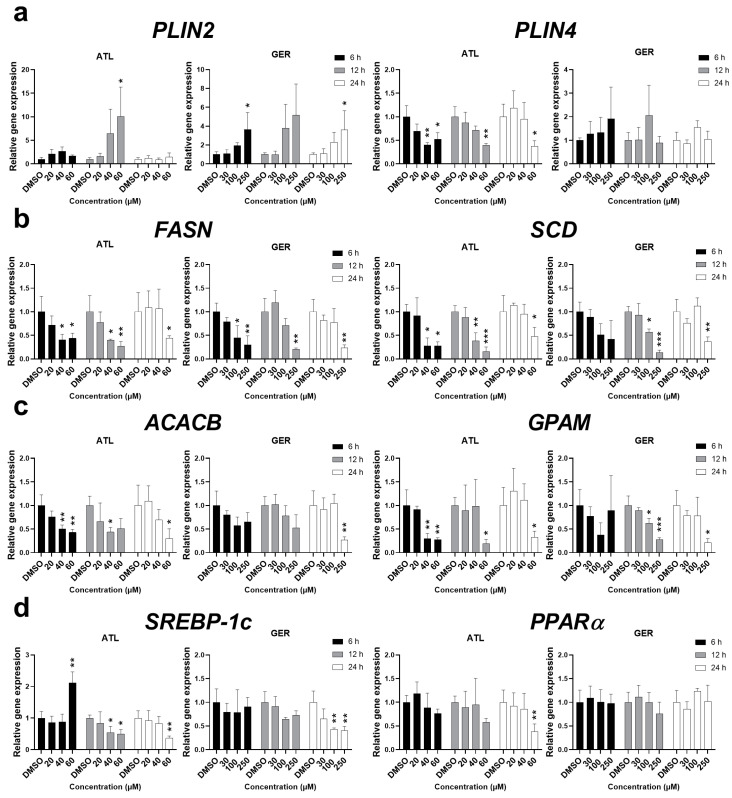
Relative gene expression changes in major genes related to (**a**) lipid sequestration, (**b**) de novo lipogenesis (**c**) triacylglyceride synthesis and (**d**) lipid metabolism regulation transcription factors, after a single exposure of ATL and GER to differentiated HepaRG cells. The relative mRNA changes were measured after 6, 12, and 24 h, using RT-qPCR. Results are expressed as the mean ± SD from three independent experiments. Results statistically significant: *p* < 0.05 (*), *p* < 0.01 (**), *p* < 0.001 (***).

To determine more information about the effect of ATL and GER on lipid metabolism in the dHepaRG cells, the effects of the sesquiterpenes on the expression of major genes related to lipid sequestration (*PLIN2, PLIN4*), de novo lipogenesis (*FASN, SCD*), and triglyceride synthesis (*ACACB, GPAM*) were studied. The mRNA of *PLIN2* was induced by both ATL and GER at the highest concentrations. On the other hand, ATL decreased the mRNA level of *PLIN4*, while no significant change was caused by GER. The mRNA levels of all the remaining genes, *FASN*, *SCD*, *ACACB,* and *GPAM*, were significantly decreased after ATL and GER treatment. Since lipid metabolism is under regulation of the transcription factors sterol regulatory element-binding protein 1c (SREBP-1c) and peroxisome proliferator-activated receptor alpha (PPARα), their gene expressions were also measured. While ATL dysregulated *SREBP-1c* mRNA and inhibited *PPARα* at the toxic concentration after 24 h, GER has inhibited *SREBP-1c* at toxic concentration after 24 h but showed no effect toward *PPARα* mRNA expression. Results of sesquiterpene-mediated changes in mRNA levels of lipid metabolism related genes are presented in Figure 6.

## 4. Discussion

The search for new cancer cures is limited by the fact that numerous anticancer agents can induce liver injury, the exact causes of which is mostly idiosyncratic and hard to discern. Therefore, alternative therapeutic agents that effectively kill cancer cells and present low or no hepatotoxicity are highly desirable [32]. Two sesquiterpenes, ATL and GER, have shown promising anticancer activity, but information about their potential hepatotoxicity is limited. For this reason, the toxicity of these interesting molecules was studied in dHepaRG cells, a hepatocyte-like model. The IC50 values for ATL and GER after 24 h were approximately 60 µM and 250 µM, respectively, with almost no change after 72 h (Table 1).

In proliferative liver (HepG2, HuH-7, LO2), breast (MCF7, MDA-MB-231), and glioblastoma cancer cell lines, their IC50 values after 12 to 24 h are approximately 10–30 µM for ATL [9,10,33,34,35] and 150–250 µM for GER [13,36,37]. Comparing these data, ATL has shown significantly lesser toxicity toward the dHepaRG cells than cancer cells, while the toxicity of GER is similar in dHepaRG cells and cancer cells. Taking ROS production into consideration, the lowest measured concentrations leading to oxidative stress in the dHepaRG cells were 20 µM ATL and 100 µM GER (Figure 1A). Similarly, these sesquiterpenes influence the mRNA expression of functionally important liver enzymes even in non-toxic/slightly toxic concentrations, especially *CYP3A4*, *G6PC,* and *TFRC* (Figure 1B). This means, that use of ALA and GER is accompanied with a potential risk of impaired liver function or induction of liver injury.

The tested compounds are known to influence the transcription factor STAT3; for ATL, it was described to target NF-κB as well. These biological activities are important, as activated NF-κB and STAT3 control the expression of anti-apoptotic, pro-proliferative, and immune response genes. Some of these genes overlap and require transcriptional cooperation between the two factors [38]. The inhibition of p-STAT3 (Y705) phosphorylation was indeed observed after ATL (all concentrations after 12-h exposure) and GER (the higher concentrations after 12 and 24-h exposure) treatment. However, the total STAT3 protein expression decrease observed in the highest concentration (equal to IC_50_) of ATL and GER (Figure 2) has not been reported previously when highly proliferative cancer cell lines were used. For example, Maryam et al. (2017) described not only the inhibition of STAT3 activation, but also the inhibition of STAT3 translocation into the nucleus as well as DNA binding ability in A549 lung adenocarcinoma cells [30]. But unlike in this research, we had already observed total STAT3 decrease in a whole cell lysate. While an NF-κB expression decrease after ATL treatment was described in other cells, an observed decrease in NF-κB expression after 12-h exposure of dHepaRG cells to GER is in contrast to the lack of observed effect of GER on NF-κB expression in breast cancer cells [16]. The transcription factor NF-κB is closely related to the RIP family of serine-threonine kinases, which are important regulators of cellular stress that triggers pro-survival and inflammatory responses through the activation of NF-κB, as well as pro-apoptotic pathways [39]. The decreased level of RIP1 caused by ATL and GER treatment of the dHepaRG cells (Figure 2A,B) showed a new biological activity that will be a topic for further research.

Both transcription factors NF-κB and STAT3 regulate countless genes and many of them together. One of these is a gene coding the intercellular adhesion molecule ICAM-1 [23,40]. ICAM-1 expression is usually low in hepatocytes, whereas expression in hepatocytes and endothelial cells can significantly increase because of extrahepatic obstructive cholestasis, especially in hepatocytes in the areas of a parenchymal injury [41,42]. ICAM-1 is more important in terms of hepatocellular carcinoma, where it is believed to be involved in metastasis [43] However, dHepaRG cells are not primary hepatocytes, but a hepatocyte-like model, and we would be interested in determining if there would be any change in its expression after sesquiterpene exposure. *ICAM-1* mRNA was significantly induced by the highest concentration of the studied sesquiterpenes (Figure 3B), whereas ICAM-1 protein, which had been expected to increase, significantly decreased after ATL (all concentrations after 12-h exposure) and GER (the higher concentrations after 12 and 24-h exposure) exposure (Figure 3A). The *ICAM-1* mRNA expression induced is usually related to pro-inflammatory conditions. Since ATL and GER decreased expression of ICAM-1 in the dHepaRG cells, there is a high probability that they will influence ICAM-1 or other adhesion molecules even in proliferative cell lines. This interaction with adhesion molecules expression could be another mechanism of the anti-cancer effects of sesquiterpene, especially if the interaction is managed to potentiate the effect without inducing toxicity towards healthy cells.

With the aim of revealing the mechanisms of the action of sesquiterpenes in the liver, the BATMAN-TCM tool was used. Out of six common targets predicted for ATL and GER (Figure S2), HMGCR, a major regulatory enzyme of cholesterol synthesis, is the most interesting, since cholesterol metabolism dysregulation is a major event in both health and disease. Our single exposure experiment showed a decrease in HMGCR level by ATL (in the higher concentrations after 12 and 24-h exposure) and by GER (the highest concentration after 12-h exposure). Most of the genes of cholesterol synthesis are under the regulation of SREBP-2 [44]. The expression of the functional SREBP-2 (F) form also showed changes in expression, but also more variability between both compounds (Figure 4). The *HMGCR* and *HMGCS* mRNA changes induced by ATL are similarly variable, depending on the measured time and concentration. In contrast to ATL, only 100 μM GER after 12 h caused significant change in *HMGCR* expression (Figure 4B). With the intention to potentiate the effect, a multiple exposure experiment was performed with the highest non-toxic concentration of sesquiterpenes. Although both sesquiterpenes induced SREBP-2 (F), only a slight decrease of HMGCR by GER was observed (Figure 5A), and no change of total cholesterol level in dHepaRG cells was observed, except for the positive control LOV (Figure 5C). Although a non-toxic concentration of ATL and GER have some effect on the mevalonate pathway, the most significant overall changes were caused by toxic concentrations.

SREBP-2 is not necessarily the only regulator of cholesterol pathway, since these genes can also be regulated by the aryl hydrocarbon receptor (AhR), based on rodent studies [45]. We have therefore selected another predicted target out of the six common targets predicted by the BATMAN-TCM: aromatase CYP19A1, which is responsible for a key step in the biosynthesis of estrogens, and we measured the *CYP19A1* mRNA expression by RT-qPCR. This enzyme was chosen because one of the major mechanisms of aromatase CYP19A1 regulation is a cross-talk between AhR and the estrogen receptor alpha (ERα) [46]. Estrogen signaling through the hepatocyte ERα was described to play a role in reverse cholesterol transport in rodent studies and represents therefore another important regulator of cholesterol metabolism [47]. It is also known that GER inhibits ERα expression as well as inhibits the expression of ERα related genes [37,48]. The tested sesquiterpenes both influenced *CYP19A1* mRNA, yet each of them in its own way. While ATL induced the expression at lower concentrations, GER decreased the mRNA level after 6 and 12 h, whereas 100 μM GER significantly induced Cyp19A1 mRNA after 24 h. This shows that each sesquiterpene may be under the effect of a different nuclear receptor, or their cross-talk varies by time and concentration, as shown by GER. Another study using HepaRG cells and the typical cytochrome P450 inducer β-naphtoflavone (BNF), a potent AhR ligand, presented AhR dependent induction of *CYP19A1* mRNA, but only in undifferentiated cells [46]. These nuclear receptors can obviously play a role in the biological activity of the mentioned sesquiterpenes and will thus be a center of interest as sesquiterpenes are further studied in both healthy and cancer cells.

GER, which has previously shown activity toward lipid metabolism, has been described as an antiadipogenic and lipolytic constituent via the regulation of adipogenesis, lipolysis and the AMP-activated protein kinase α (AMPKα) pathway in 3T3-L1 preadipocytes [27]. In high fat diet mice, attenuation of hyperlipidemia and improved lipid metabolism were suggested by suppressed fatty acid synthesis and uptake by inhibiting SREBP-1 and 2 signaling activation along with improved lipid metabolism [26]. In this study, no further modifications such as lipid overload with free fatty acids to the dHepaRG cells were performed. Despite this, significant changes in the mRNA transcripts of genes involved in lipid metabolism were observed during our single exposure experiment. *PLIN2* and *PLIN4* genes are coding proteins responsible for the formation of lipid droplets, and in the liver, these genes are direct targets of PPARα [49,50]. The other genes related to fatty acid and triglyceride synthesis showed decreased levels after sesquiterpene exposure at higher concentrations. Measuring the expression of *SREBP-1c* and *PPARα* mRNA has shown that ATL influences both transcription factors, while GER influenced only *SREBP-1c*. This suggests that studied sesquiterpenes ATL and GER have differences in their mechanism of action targeting the lipid metabolism, although the final phenotype is very similar (Figure 6).

## 5. Conclusions

In conclusion, the sesquiterpenes ATL and GER significantly decreased protein levels of transcription factors STAT3 and NF-κB as well as their transcription target ICAM-1 in dHepaRG cells. Both compounds exerted considerable pro-oxidative potential, as they increased ROS production even at non-toxic concentrations. Moreover, ATL and GER exerted dysregulating effects toward cholesterol and lipid metabolism, during which these compounds influenced the protein expression of HMGCR and SREBP-2 as well as mRNA expression of *HMGCR*, *CYP19A1*, *PLIN2*, *FASN*, *SCD*, *ACACB,* and *GPAM*. In view of these results, the use of ATL and GER as potential anticancer agents seems to be disputable, as these compounds influenced the viability and metabolism of non-cancerous cells in concentrations equal to or very close to the IC_50_ values obtained for cancerous cells.

## Figures and Tables

**Table 1:** Concentrations of ATL and GER (μM) resulting in 50% inhibition of dHepaRG cell viability (IC50) after 24 and 72-h single exposure, measured by MTT assay and lactate dehydrogenase (LDH) leakage. Examples of previously reported IC50 values for proliferative liver, breast, and glioblastoma cell lines are given.

Time (h)	Alantolactone IC_50_ ± SD (µM)		Germacrone IC_50_ ± SD (µM)		Previously Reported IC_50_ Values (24 h)	
	MTT	LDH	MTT	LDH	Alantolactone	Germacrone
24	58.9 ± 3.5	61.1 ± 1.8	255.7 ± 5.7	243.6 ± 15.5	HepG2: 7 µM [35], 30 µM [36], 33 µM [9]; MCF-7: 36 µM [14]	HepG2: 160 µM [12], 170 µM [7], >240 µM [13]; MCF7: 195 µM
72	57.2 ± 1	57.8 ± 2	242.3 ± 17.4	234.2 ± 13.4	MDA-MB-231: >15 µM [8]; U87: 20 µM [10], 33 µM [34]	[11], >200 µM [37]; MDA-MB-231: 244 µM [11]; U87: 175 µM [36]

## Supplementary Materials

The following are available online at https://www.mdpi.com/2072-6643/12/6/1720/s1, Figure S1: Effects of ALA and GER on the viability of dHepaRG cells measured by A) MTT assay and B) LDH assay after 24 and 72 h., Figure S2: Target prediction analysis of alantolactone and germacrone by BATMAN-TCM. Venn diagrams represent the number of targets predicted for each sesquiterpene and intersection represents common targets for both sesquiterpenes, Table S1: List of primary antibodies for western blot, Table S2: List of primers for RT-qPCR, Table S3: Results from BATMAN-TCM by input compounds denoted by PubChem CIDs.
