# Supplementary material for: The Hepatotoxicity of Alantolactone and Germacrone: Their Influence on Cholesterol and Lipid Metabolism in Differentiated HepaRG Cells
Source: Nutrients. 2020 Jun 8;12(6):1720. doi: 10.3390/nu12061720 (PMC7353089; doi:10.3390/nu12061720)
Supplement: Supplementary file 1 [file nutrients-12-01720-s001.pdf]

Supplementary data

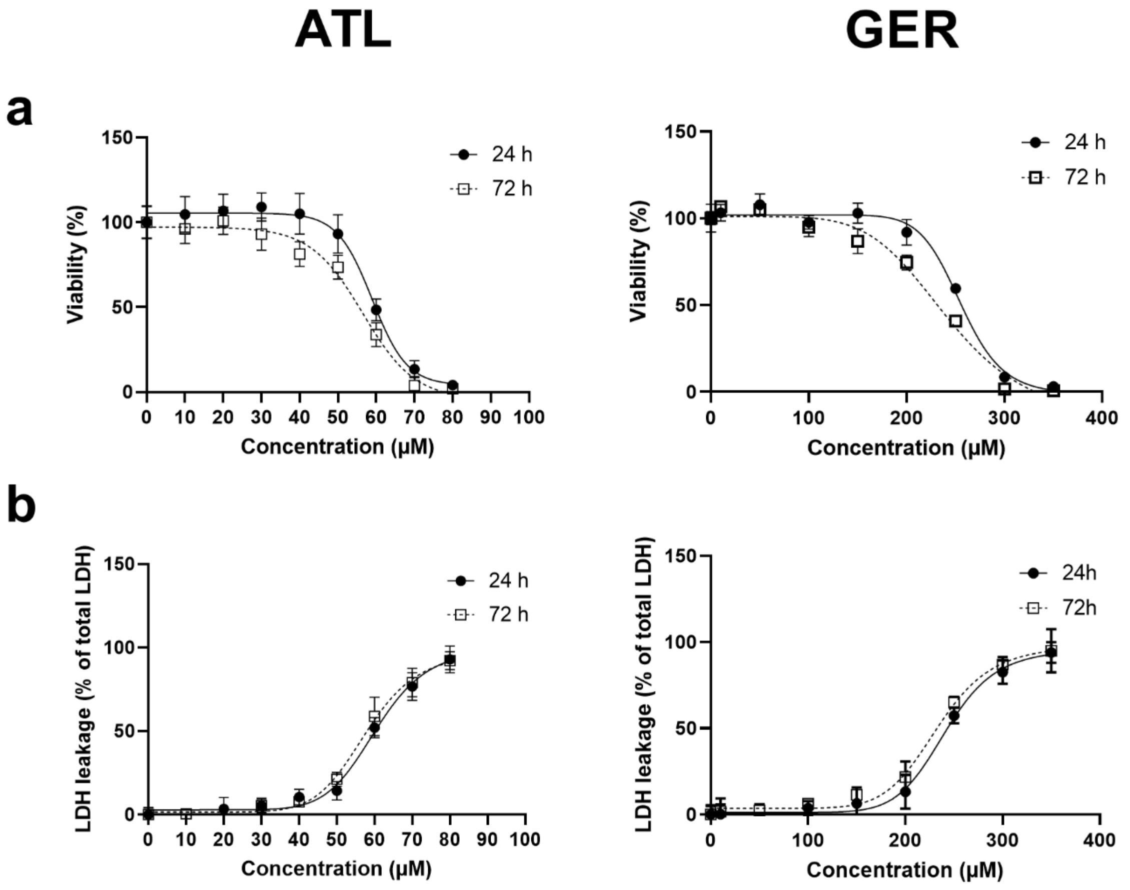

Supplementary Figure 1: Effects of ATL and GER on the viability of dHepaRG cells measured by a) MTT assay and b) LDH assay after 24 and 72 h.

Supplementary Table 1: List of primary antibodies for western blot.

Antigen	Catalog number	Dilution	Source	Supplier
NF-κB	ab32536	1:50000	Rabbit	Abcam
RIP1	D94C12	1:1000	Rabbit	Cell signalling
STAT3	ab68153	1:1000	Rabbit	Abcam
p-STAT3 (Y705)	ab76315	1:1000	Rabbit	Abcam
ICAM-1	4915	1:5000	Rabbit	Cell signalling
HMGCR	ab174830	1:2000	Rabbit	Abcam
SREBP-2	ab30682	1:300	Rabbit	Abcam
β-actin	ab8227	1:2500	Rabbit	Abcam

Supplementary Table 2: List of primers for RT-qPCR.

Gene symbol	Gene name	Accession Number	Forward	Reverse
SDHA	Succinate dehydrogenase complex, subunit A	NM_004168.3	TGGGAACAAGAGGGCATCTG	ACCACCACTGCATCAAATTCATG
YWHAZ	Tyrosine 3-monooxygenase/tryptophan 5-monooxygenase activation protein zeta	NM_003406.3	TGATCCCCAATGCTTCACAAG	GCCAAGTAACGGTAGTAATCTCC
CYP3A4	Cytochrome P450 3A4	NM_017460.6	CCCCTGAAATTAAGCTTAGGAGG	CTGGTGTTCTCAGGCACAGA
A1AT	Alpha-1-antitrypsine	NM_001127700.2	TGGAAAATGAACTCACCCACG	CCAGGACGCTCTTCAGATCA
ALB	Albumin	NM_000477.7	TGTTTCACGAGCTCAACAAGT	ACGTTCCCAAAGAGTTTAATGCT
G6PC	Glucose-6-phosphatase catalytic subunit	NM_000151.4	ACTTGCTCCAAATACCAGTGC	GCCGCACAAGAAGTCGTTG
TFRC	Transferrin receptor	NM_003234.4	CATTTGTGAGGGATCTGAACCA	GACACGATCATTGAGTTTCTTCA
SCD	Stearoyl-CoA desaturase	NM_005063.5	GGAGGAGATAAGTTGGAGACGA	AGACATAAGGATGATGTTTCTCCAG
FASN	Fatty acid synthase	NM_004104.5	CGCTCTGGTTCATCTGCTCT	ATGGAATCTCGGAAGCGGTC
ACACB	Acetyl-CoA carboxylase-beta	NM_001093.4	GAACGTGCATGACAGATTTCTT	GCCATTCATGATGAGAACGA
GPAM	Glycerol-3-phosphate acyltransferase	NM_001244949.2	TGTTGTATGGACATTCTGCAC	AAACATCTATTGTACCAAGGGTCA
PLIN2	Perilipin 2	NM_001122.4	TTCTATGGCCATTGTGTTGC	ACGCCTTTTCAGATCACACC
PLIN4	Perilipin 4	NM_001367868.1	CAGAACTGGTTACCTAGTACCC	TACACTGAGCACATCCGGG
SREBP-1c	Sterol regulatory element-binding protein	AB373959.1	GCACTGAGGCAAAGCTGAAT	ACAATAGTCAAAAGCGGGGTG
PPARα	Peroxisome proliferator-activated receptor alpha	NM_005036.6	CGGCGAATTATGCTCAATGG	ACAATAGTCAAAAGCGGGGTG
HMGCR	3-hydroxy-3-methylglutaryl-coenzyme A reductase	NM_000859.2	ACCCTCGATGCTCTTGTTGA	CCCCTGACATGGTACCAACT
HMGCS	Hydroxymethylglutaryl-CoA synthase	NM_001098272.3	AACCTTTCCTATGATTGCATTG	TCCATAGCATGCATTAGTTGTG
SOAT1	Sterol O-acyltransferase 1	NM_003101.6	ATCCGCTGATCCGTTCTCTC	AGGCACGTTCTCTCTGACAA
CYP19A1	Aromatase	NM_000103.3	GCTATGTGGACGTGTTGACC	TTGATGAGGAGAGCTTGCCA
ICAM-1	Intercellular Adhesion Molecule 1	NM_000201.2	CCGCAGTCATAATGGGCACT	AGGCGTGGCTTGTGTGTT

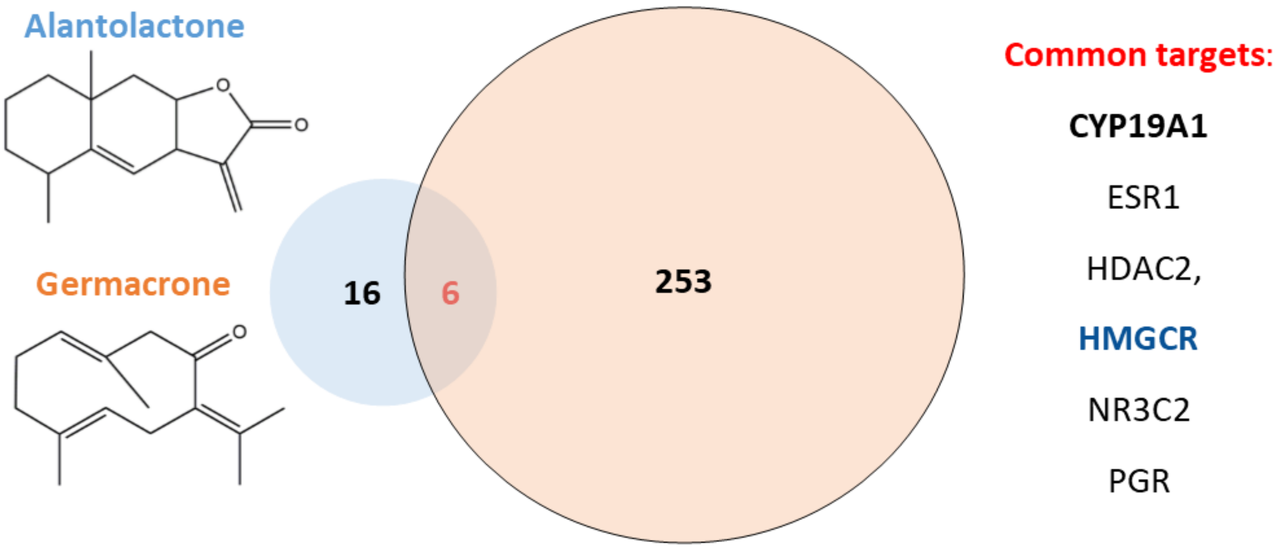

Supplementary Figure 2. Target prediction analysis of **alantolactone** and **germacrone** by BATMAN-TCM. Venn diagrams represent the number of targets predicted for each sesquiterpene and intersection represents common targets for both sesquiterpenes.

Supplementary Table 3: Results from BATMAN-TCM by input compounds denoted by PubChem CIDs.

Compound (PubChem CID)	Predicted targets (score given by target prediction method)
Alantolactone (72724)	CHRM3(80.882), CHRM1(80.882), CHRM2(80.882), ESR1(48.000), PGR(48.000), AR(48.000), NR3C1(48.000), HMGCR(48.000), ITGB2(48.000), ITGAL(48.000), CYP19A1(48.000), HDAC2(48.000), NR3C2(48.000), ARFGEF2(22.373), DOCK5(22.373), NTSR1(22.373), CHRM5(22.373), GNA15(22.373), P2RX1(22.373), DOCK4(22.373) MAP2K1(22.373), CHRM4(22.373)
Germacrone (6436348)	PDE7B(80.882), PDE5A(80.882), PDE9A(80.882), PDE4A(80.882), RYR1(80.882), CYP17A1(80.882), PDE3A(80.882), ADORA2A(80.882), ITPR1(80.882), PDE6A(80.882), PRKDC(80.882), PIK3CD(80.882), ESR1(80.882), PDE3B(80.882), PGD(80.882), PIK3CA(80.882), ADORA2B(80.882), PDE1A(80.882), PDE4D(80.882), PGR(80.882), PIK3CB(80.882), ITPR2(80.882), PDE1B(80.882), OPRK1(80.882), PDE7A(80.882), ITPR3(80.882), POLA2(80.882), PDE4C(80.882), PDE10A(80.882), PDE1C(80.882), ATM(80.882), PDE6B(80.882), PDE4B(80.882), PDE2A(80.882), ADORA1(80.882), CYP19A1(80.882), PDE8B(80.882), NT5E(80.882), HDAC2(80.882), PDE6C(80.882), PDE11A(80.882), PDE8A(80.882), NR3C2(80.882), RINT1(55.444), RIPK1(55.444), PIK3R1(55.444), WNT4(55.444), TACR2(55.444), AMPD3(55.444), CX3CR1(55.444), ADA(55.444), HAP1(55.444), IDNK(55.444), HDAC1(23.000), TFAP2C(22.373), HMGA2(22.373), TNFSF11(22.373), NT5C1A(22.373), ACTN3(22.373), NOS1AP(22.373), TAC1(22.373), PHB(22.373), SIRT1(22.373), DGKI(22.373), FKBP1A(22.373), FURIN(22.373), TOX3(22.373), UTS2(22.373), STUB1(22.373), CTR9(22.373), G6PD(22.373), GNAT1(22.373), SIX1(22.373), QDPR(22.373), TGFB1(22.373), ATP11C(22.373), KHDRBS1(22.373), PPP3CB(22.373), SYT2(22.373), SIX4(22.373), SIRT2(22.373), SPI1(22.373), HOMER1(22.373), SHANK3(22.373), TAS1R1(22.373), TRIM28(22.373), XRCC4(22.373), MED1(22.373), NKX3-1(22.373), UBE2B(22.373), ESR2(22.373), HCN4(22.373), EDN1(22.373), CACNA1A(22.373), STAT5A(22.373), TP53(22.373), GNAT3(22.373), HELB(22.373), COMT(22.373), SLC26A6(22.373), ADRA2A(22.373), YWHAE(22.373), CETN2(22.373), POLA1(22.373), CRP(22.373), CETN1(22.373), NUDT9(22.373), PIK3CG(22.373), BMP2(22.373), TNF(22.373), NEDD4(22.373), AGT(22.373), FER(22.373), SHH(22.373), SERPINB3(22.373), FOXP3(22.373), CXCL13(22.373), ARID1A(22.373), FGF10(22.373), KCNB1(22.373), LEF1(22.373), PTK2B(22.373), LIG4(22.373), ADRBK1(22.373), MAOB(22.373), SORCS3(22.373), ADRB1(22.373), ADAM8(22.373), VTI1A(22.373), SLC44A4(22.373), ADAP2(22.373), LRRC8A(22.373), FGFR2(22.373), AURKA(22.373), MCM3(22.373), VCP(22.373), GHRL(22.373), BCL11B(22.373), BCL2(22.373), 8-Mar(22.373), GAS6(22.373), C5(22.373), SREBF1(22.373), DRD2(22.373), PLN(22.373), DNM3(22.373), CHRNA3(22.373), TCF3(22.373), SRC(22.373), LONP1(22.373), XCL1(22.373), HSP90AB1(22.373), HIBADH(22.373), STAP1(22.373), HRC(22.373), UTS2R(22.373), SLC6A4(22.373), HMGCR(22.373), HIPK2(22.373), PPP3CA(22.373),

	RAPGEF2(22.373), TET1(22.373), C2CD5(22.373), CSF2(22.373), TALDO1(22.373), BDKRB2(22.373), KCNH2(22.373), MMP28(22.373), HIF1A(22.373), KIT(22.373), INS(22.373), MTOR(22.373), WNT10B(22.373), SLC18A3(22.373), ABCC4(22.373), BNIP3(22.373), ZFPM1(22.373), GATA3(22.373), UBR5(22.373), AREG(22.373), CRH(22.373), ZFP42(22.373), PIK3R6(22.373), PF4(22.373), ATP1A2(22.373), AVPR2(22.373), CAMK2D(22.373), ADRA1B(22.373), IL10(22.373), NEFL(22.373), UCN2(22.373), ATP1A1(22.373), RYR3(22.373), PPP3R1(22.373), SPX(22.373), ATP2A1(22.373), SMO(22.373), RAG1(22.373), RAB8B(22.373), DKK3(22.373), AHCYL1(22.373), AQP1(22.373), TNFAIP3(22.373), TAC4(22.373), DPPA3(22.373), CASQ1(22.373), FKBP1B(22.373), CDC42(22.373), IL4(22.373), POLB(22.373), IDO1(22.373), ATP2B4(22.373), CTNNB1(22.373), PGLS(22.373), SLC9A3R1(22.373), IGF2(22.373), HPS4(22.373)TKT(22.373)DRD1(22.373)ADAP1(22.373) EDA(22.373), RYR2(22.373), CD34(22.373), HSP90AA1(22.373), LTA(22.373), SPR(22.373), GPER1(22.373), CBFA2T3(22.373), CALHM1(22.373), WNT5A(22.373), SELP(22.373), XRCC6BP1(22.373), PARP10(22.373), HPRT1(22.373), AMICA1(22.373), TAS1R3(22.373), NOS1(22.373), TREM1(22.373), TAL1(22.373), ALAD(22.373), PAWR(22.373), SOX9(22.373), BMP5(22.373), PDGFB(22.373), RTN2(22.373), CD63(22.373), CAMK2G(22.373), LAMP2(22.373), NEFH(22.373), GLYR1(22.373)
